# Strength Back – A qualitative study on the co-creation of a positive psychology digital health intervention for spinal surgery patients

**DOI:** 10.3389/fpsyg.2023.1117357

**Published:** 2023-04-21

**Authors:** Annemieke Y. Van Der Horst, Ernst T. Bohlmeijer, Karlein M. G. Schreurs, Saskia M. Kelders

**Affiliations:** ^1^Centre for eHealth and Wellbeing Research, Faculty of Behavioural, Management and Social Sciences, University of Twente, Enschede, Netherlands; ^2^Research Centre Smart Health, Saxion University of Applied Sciences, Deventer, Netherlands; ^3^Roessingh Research and Development, Enschede, Netherlands; ^4^Optentia Research Focus Area, North-West University, Vanderbijlpark, South Africa

**Keywords:** positive psychology, acceptance and commitment therapy, spinal surgery, digital health intervention, co-creation

## Abstract

**Introduction:**

Spinal surgery patients often experience pain as well as stress, anxiety or even depression before surgery, highlighting the need for better mental preparation before undergoing surgery. Acceptance and Commitment Therapy and positive psychology have proven effective in coping with chronic pain and providing long-term skills that enhance psychological flexibility and mental well-being.

The aim of this study is to develop a digital intervention (app) based on Acceptance and Commitment Therapy and positive psychology in co-creation with all stakeholders, including patients and professionals. The aim of the intervention is to increase psychological flexibility and positive skills of spinal surgery patients to promote long-term resilience.

**Materials and methods:**

In this qualitative study, individual, semi-structured interviews were held with healthcare professionals (*N* = 9) and spinal surgery patients (*N* = 12) to identify contextual factors and needs for the app. Subsequently, three focus-group sessions were held with healthcare professionals and newly recruited patients to specify relevant values. Also, a first version of the app, named Strength Back, was developed using a participatory design.

**Results:**

The interviews confirmed the need for information and digital support to cope with insecurity, anxiety and pain, both before and after surgery. Based on iterative steps in the focus-group sessions, thirteen modules were developed focusing on procedural information, pain education, psychological flexibility and mental well-being.

**Discussion:**

The intervention Strength Back, containing information as well as Acceptance and Commitment Therapy and positive psychology exercises, has the potential to increase psychological flexibility, enhance well-being and improve postoperative recovery after spinal surgery.

## 1. Introduction, background, and purpose

### 1.1. Introduction

Spinal surgery patients often experience high levels of pain before surgery and there is no guarantee this pain will be alleviated completely after surgery (Hoffman et al., 1993; Beauregard et al., 1998; Thomson, 2013; Hoofwijk et al., 2015). In addition, these patients experience stress, anxiety or even depression before surgery (van der Horst et al., 2019). This warrants better mental preparation before surgery and a long-term focus for perioperative interventions to enable patients to deal with surgery resistant or recurrent pain. Acceptance and Commitment Therapy and positive psychology have proven effective in coping with chronic pain and providing long-term skills that enhance psychological flexibility and mental well-being (A-Tjak et al., 2015; Veehof et al., 2016; Hughes et al., 2017; Peters et al., 2017; Simpson et al., 2017). Such long-term skills might also benefit spinal surgery patients.

This qualitative study describes the development of a digital health intervention (app) based on Acceptance and Commitment Therapy and positive psychology in co-creation with all stakeholders, including patients and professionals. The aim of the intervention is to increase psychological flexibility and positive skills of spinal surgery patients to promote long-term resilience.

### 1.2. Background

Spinal surgery patients report high levels of physical complaints as well as insecurity, pain, stress and anxiety, before deciding to undergo surgery (van der Horst et al., 2019). Unfortunately, there is no guarantee that surgery will resolve all issues; postsurgical recovery entails moderate to severe postoperative pain for 40–60% of patients (Hoffman et al., 1993; Beauregard et al., 1998; Thomson, 2013; Hoofwijk et al., 2015). In addition, about 20–30% of spinal surgery patients do not experience (long-term) improvement in pain relief after surgery (JA et al., 1992; Hoffman et al., 1993; Inoue et al., 2017; Weir et al., 2017). This results in a longer hospital stay, longer physical and mental recovery, delayed return to work, higher healthcare costs and the potential development of chronic pain. The potential transition from postoperative pain into chronic pain is a major issue, because chronic pain affects many aspects of a patient’s life including work, physical, emotional and social well-being, and quality of life (Walker et al., 2006; Duenas et al., 2016).

The experience and intensity of perioperative and chronic pain is not only dependent on physical aspects, but is heavily influenced by the cognitions, emotions and expectations of patients. The Fear Avoidance model explains the trajectory from acute to chronic pain, through fear and catastrophizing, the tendency to enlarge the threat of pain and a feeling of helplessness, leading to an increase in pain avoidance as dominant coping strategy (Vlaeyen and Linton, 2012). In turn, pain avoidance leads to a less active lifestyle, thereby worsening instead of relieving pain. Hasenbring & Verbunt elaborated on this model by adding a pathway of endurance coping with pain in their Avoidance-Endurance Model (Hasenbring and Verbunt, 2010). Whereas avoidance leads to passive behaviour, endurance coping leads to forced activity and a suppression of pain signals, perpetuating the pain. The Fear Avoidance model and the Avoidance-Endurance model can also be applied to pre- and postoperative pain: fear and high levels of catastrophizing have been found to predict higher levels of (postoperative) pain, pain chronicity and reduced quality of life (Pavlin et al., 2005; Khan et al., 2011; Hoofwijk et al., 2015). Also, unrealistic or unfulfilled expectations about surgery and preoperative stress may lead to the experience of higher levels of postoperative pain (Iversen et al., 1998, 2015; Munafo and Stevenson, 2003; Arpino et al., 2004; Granot and Ferber, 2005; Morone et al., 2010; Mancuso et al., 2016).

Because of the large role of cognitions, emotions and expectations of patients in the experience and intensity of perioperative and chronic pain, psychological interventions may be useful. Powell et al. (2016) reviewed several studies and found that psychological techniques such as procedural information; sensory information; behavioural instruction; cognitive intervention; relaxation techniques; hypnosis and emotion focused interventions are all associated with lower postoperative pain, shorter length of hospital stay and reduced negative affect, compared to control groups (Powell et al., 2016). However, they concluded that there is currently a lack of strong evidence for the beneficial role of psychological preparation due to poor reporting and high levels of heterogeneity in types of surgery, interventions and outcomes. A possible explanation for this lack of strong evidence might be that most current psychological preparation techniques before surgery focus on reducing the negative affect (e.g., anxiety and depression). There is also evidence that coping strategies such as (pain) acceptance, engaging in beneficial social interactions and experiencing a value-based purpose in life are more appropriate for improving mental well-being and promoting resilience in the face of (chronic) pain (Smith and Zautra, 2004; Sturgeon and Zautra, 2010). A second limitation of current psychological preparation techniques is their primary focus on reducing distress in the short-term, i.e., the period before the operation, whilst Sturgeon & Zautra (Sturgeon and Zautra, 2010) argued that sustainable resilience to chronic pain also requires skills promoting adaptation and mental health in the long term.

To overcome these limitations, it may be worthwhile to investigate whether other approaches may be of value in the psychological preparation for spinal surgery with the goal of reducing perioperative and chronic pain. Two promising options that focus on skills useful for the longer term are Positive Psychology (Seligman and Csikszentmihalyi, 2000) and Acceptance and Commitment Therapy (Hayes et al., 2012). Positive Psychology (PP) is the scientific study of well-being and optimal functioning, focusing on human flourishing instead of reducing risk-factors for psychopathology and malfunctioning. PP involves topics as strengths, virtues, meaning, happiness, gratitude, compassion, resilience and flourishing (Bohlmeijer et al., 2021). Mental well-being is defined as a state of happiness and contentment, with low levels of distress and a good quality of life. Mental well-being comprises positive emotional, psychological and social functioning. The presence of higher levels of these three dimensions of well-being is an indicator of flourishing (Keyes, 2005, 2007; Westerhof and Keyes, 2010). Positive psychology interventions (PPIs) aim to promote positive resources and skills that contribute to successful adaptation and mental health (Bohlmeijer and Westerhof, 2021). Acceptance and Commitment Therapy (Hayes et al., 2012) is based on the relational frame theory and focuses on performing value-based activities in life, even in the face of insecurity and adversity. The aim of treatment is to increase psychological flexibility which in the context of pain, implies that painful sensations, feelings and thoughts are accepted, as opposed to avoided, and that attention is shifted toward personally valued goals (McCracken and Vowles, 2014). Pain acceptance in ACT refers to the capacity to continue with life even in the face of pain, instead of fighting or trying to control the pain and letting it interfere with daily functioning.

PPIs and ACT have been found effective in the treatment of chronic pain (A-Tjak et al., 2015; Veehof et al., 2016; Hughes et al., 2017; Peters et al., 2017; Simpson et al., 2017) and in improving affect and functional ability after knee surgery (Smith and Zautra, 2004). As this positive approach that focuses on gaining skills for the longer term seems to work for pain patients, it could also be beneficial for spinal surgery patients in preparing them for surgery and reducing perioperative and chronic pain.

PPIs and ACT are usually offered in face-to-face settings, but more and more, they are offered online through digital interventions. Advantages of digital self-help interventions are that they can be accessed anytime and anywhere. In addition, these interventions can be tailor-made for specific patient populations and can include several forms of information (e.g., video or text). Moreover, they can be effective: Bolier and Abello (Bolier et al., 2013) reviewed digital PPIs and found that the majority of the included studies showed improvement in well-being or reduction of distress in the intervention group, compared to the control group.

Also in the context of medical healthcare and in particular perioperative care, digital interventions seem promising. Austin et al. (2020) found, particularly with online compassion-based interventions, that people with long-term medical conditions experienced benefits regarding the acceptance of the condition, improved emotion regulation skills, reduced feelings of isolation and reductions in depression and anxiety compared to control groups. Other studies have indicated an improvement in outcomes for patients using web-based interventions, compared to only having face-to-face interventions (Wantland et al., 2004; van der Meij et al., 2016). Wantland et al. (2004) found that the use of web-based interventions led to increased exercise time, increased knowledge, increased participation in healthcare and slower health decline for patients with a chronic illness. For patients undergoing surgery, Van der Meij et al. (2016) found that in the majority of the studies in their review e-health led to similar or improved clinical patient-related outcomes compared to only face-to-face perioperative care. Similarly, Knight et al. (2021) reviewed digital health interventions measuring several patient outcomes after surgery. Their results indicate that digital health interventions have the potential to reduce complication rates, facilitate patient recovery, reduce inappropriate service use and improve longer-term outcomes after surgery. Interestingly, they also state that the studies in their review made little reference to engaging patients in the development of the digital health interventions. They see this as a missed opportunity and encourage future patient-centred research and interventions as this might also mitigate the problem of patient attrition from digital health interventions (Knight et al., 2021).

Although digital self-help interventions can have positive effects, to optimise their benefits it is thus vital that they are systematically developed in a process of co-creation with stakeholders and patients (Klein et al., 2014; Scott et al., 2017; Knight et al., 2021). To develop and implement a high-quality digital intervention, it is necessary to include the experiences, needs and wishes of the intended users. The CeHRes (Centre for eHealth Research and Disease Management) Roadmap (van Gemert-Pijnen et al., 2011) serves as a guideline for eHealth development, implementation and evaluation. It is a holistic framework aimed at improving the uptake and impact of eHealth technologies by involving stakeholders right from the start and by continuous, formative evaluation. The framework consists of several phases, starting with a contextual inquiry and value specification, before designing, operationalising and evaluating the intervention (van Gemert-Pijnen et al., 2011). The framework has been successfully used to develop effective digital interventions (Kelders et al., 2013; Kip et al., 2019).

### 1.3. Purpose

In summary, there is a need for psychological interventions for spinal surgery patients supporting their long-term recovery. Ideally these interventions should start before the operation, continue afterwards and have a positive approach. However, there is a current lack of such interventions. The purpose of this study was to develop a digital self-help intervention (app) based on PP and ACT in co-creation with all stakeholders, i.e., patients and professionals. We aim to answer the following research question: How can a digital health intervention based on positive psychology and Acceptance & Commitment Therapy be developed in co-creation with spinal surgery patients and healthcare professionals? Contextual inquiry and value specification were conducted to determine important needs and contextual factors relating to a supportive digital intervention. An app was then designed based on the focus-groups and interviews. Future researchers developing an intervention could benefit from this process, because we describe in detail the different steps undertaken, using the holistic framework of the CeHRes Roadmap.

## 2. Materials and methods

### 2.1. Study design

**Figure 1:**
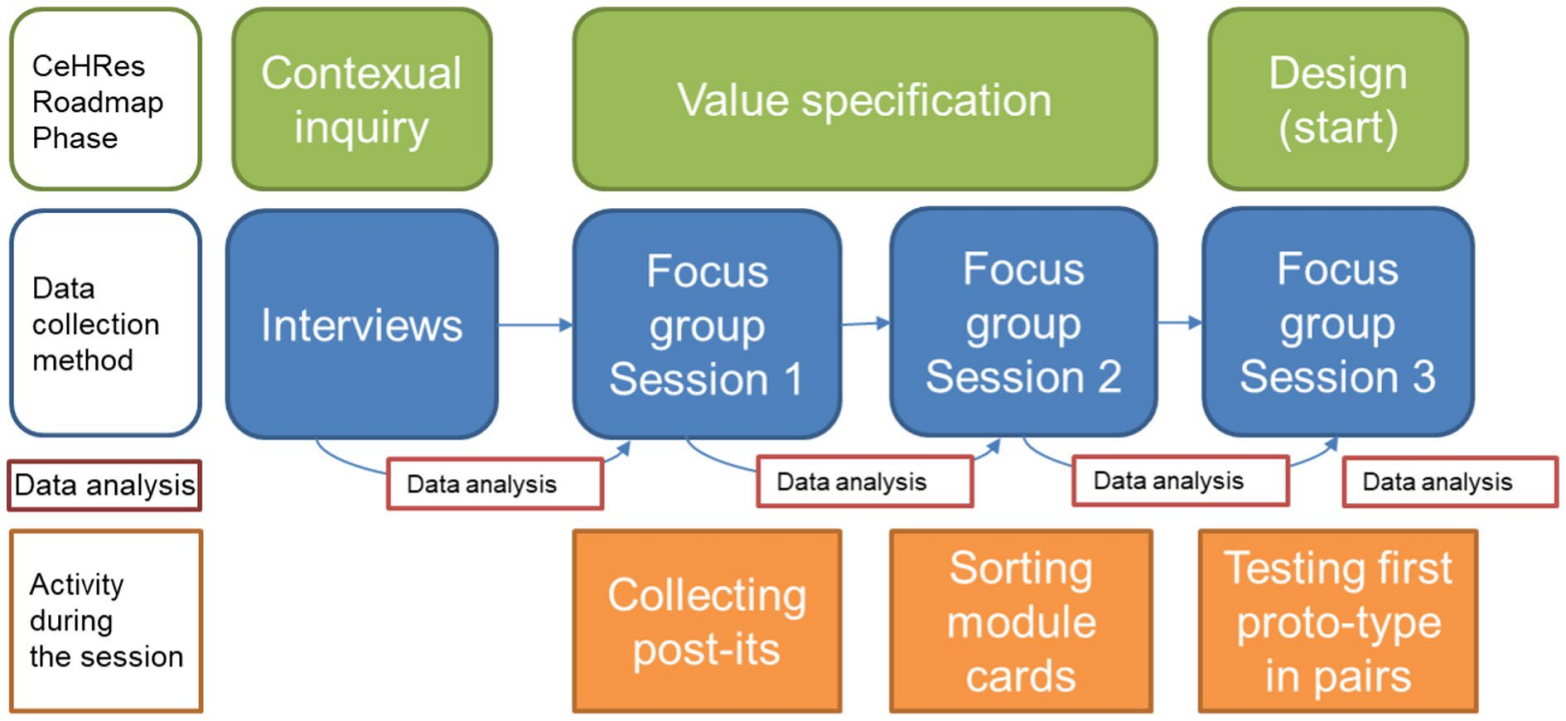
Overview of the study design and used methods.

The study overview and used methods are shown in Figure 1. We conducted a qualitative research study, using a participatory design approach (Simonsen and Robertson, 2012). Participatory design implies that not only input from future users should be used, but that stakeholders should be included in the developmental process as co-designers and meaningful participators throughout the entire design process (Sanders and Stappers, 2008; Simonsen and Robertson, 2012; Jessen et al., 2020). As a first step in the current study, the input for the developmental process was gathered in individual interviews. This limited the potential bias of groupthink and desirability. In addition, it enabled both patients as well as professionals to voice their personal needs and preferences in a safe and personal setting. In the subsequent focus group sessions the aim was to not only gather more input from the different stakeholders, but also to include them as co-designers. This design is therefore in line with the principles of participatory design as described by Simonsen and Robertson (2012).

In addition, to ensure crucial stakeholder involvement, the CeHRes Roadmap (van Gemert-Pijnen et al., 2011) was used to develop a digital health intervention. The focus of the current article is on the first two phases of the roadmap (see Figure 1): contextual inquiry and value-specification. Both individual interviews and focus-group sessions were conducted with patients and professionals to determine important needs and contextual factors relating to a supportive digital intervention. The input generated from these two phases was used to develop a first prototype of the intervention, thereby initiating the third phase of the roadmap: design.

### 2.2. Sub-study 1: Interviews with patients and professionals

#### 2.2.1. Procedure and recruitment

Patients were recruited at an orthopaedic surgery centre in the Netherlands. They had all undergone spinal fusion or decompression surgery in the previous 6 months and were at least 18 years of age. As this is already quite a specific target group, we have decided not to focus on a subgroup of these patients, for example differentiating in length of time diagnosis or disease severity.

Eligible patients received a letter at home informing them about the study and asking them to participate. A week later the researcher called them, suggesting a meeting for the interview. Seven patients were included. These participants were predominantly older adults and retired from work. As the context, needs and wishes and values of retired patients might differ from working patients, five more patients were included, including younger patients. In total 12 patients agreed to an interview (*n* = 12).

Professionals (*n* = 9) were recruited at an orthopaedic surgery centre in the Netherlands. Two orthopaedic surgeons, a physical therapist and a nurse practitioner were approached *via* email and all agreed to take part in an interview. A nursing supervisor was contacted by email to recruit nurses for the interviews. The supervisor then contacted the researcher with the names of 5 nurses who had agreed to take part. Subsequently these nurses were emailed and all of them participated in an interview.

#### 2.2.2. Interviews and materials

Semi-structured interviews were conducted by the first author (AH) between August 2015 and December 2016. These interviews were held at the participants’ home (patients) or the orthopaedic centre (professionals) and were audio recorded. At the start of the interview, written informed consent was obtained from all participants. Interviews lasted 50–90 min, the majority lasting around 60 min. After the interview participants received a small gift to thank them for their time and effort.

The interviews were part of a broader study into the experiences of spinal surgery patients and have been published elsewhere (van der Horst et al., 2019). For the current study, only the last part of the interview scheme was used. This part focused on their opinions regarding a supportive digital health intervention and had not been used in the earlier publication. Participants were asked whether they thought a digital intervention could have helped patients during their experiences both before and after surgery. Furthermore, they were asked about their needs and wishes for such an intervention. Examples of questions: Would you use a digital health intervention, why (not)? How might a digital health intervention have helped you in the time before and after surgery? What kind of elements should a digital health intervention for spinal surgery patients contain? In addition, several questions on timing, intensity and preferred guidance for the intervention were asked. The professionals were also asked whether they were willing to provide any type of support or guidance to patients during their use of the intervention. For both participant groups, demographic characteristics were also noted.

#### 2.2.3. Data analysis

Thematic Analysis (Braun and Clarke, 2006) was used to analyse the data. After recording, the interviews were transcribed verbatim by one author (AH). Following transcription, the interviews were read and re-read by one author (AH) in order to become familiarised with the data.

For the coding and analysis process Atlas.ti software (version 9) was used. The coding process was performed by one author (AH) followed an inductive approach (Ritchie et al., 2003). As a result of this process a primary code book was generated, which was supplemented during the entire, iterative coding process. This process of open coding continued until all interviews were coded. No new codes were generated in the last 5 interviews, suggesting saturation. All codes and themes were reviewed by two authors (AH and SK) and defined. The names of the themes were slightly adjusted, ensuring they fully represented the data.

### 2.3. Sub-study 2: Focus-groups with patients and professionals

#### 2.3.1. Procedure and recruitment

Patient participants had all undergone a spinal fusion or decompression surgery in the previous 6 months and were at least 18 years of age. An orthopaedic surgeon screened patients for eligibility after which the researcher contacted these patients. Eligible patients (*n* = 10) received a letter at home informing them of the study and asking them to participate. Two of the approached patients replied that they did not want to participate. The researcher called the remaining participants (*n* = 8), asking them to participate in the focus-groups. Two patients stated on the phone they did not want to participate. All other selected patients (*n* = 6) agreed to participate in the focus-groups. One of these patients withdrew from participation, resulting in 5 patients participating in the first focus-group session. All 5 participants were invited to the second session, but only two attended. The other three participants did not respond to the invitation. To ensure patient participation in this process, 5 new eligible patients were approached before the third session. We were unable to contact one patient, one was unavailable on the session date and one did not wish to participate. The remaining two new patients took part in the third session, together with two previous patients, resulting in a total of 4 patients participating in the third session.

Professionals participating in the focus-groups were recruited by email. Several types of healthcare professionals were approached by email (orthopaedic surgeons, physical therapist, nurse practitioner, research coordinator) or by emailing their team leader (nurses). All professionals who were approached agreed to take part in the focus-groups and did so for at least one session. The orthopaedic surgeon, physical therapist, nurse practitioner and one of the nurses had also participated in the previously held interviews. Other professionals were new to the project. See Appendix B for a total overview of participants in the focus-group sessions.

#### 2.3.2. Focus-groups and materials

The focus-groups were held in three sessions. The sessions were conducted by one researcher (AH) in March, April and May in 2019, with one research-assistant taking notes of key participant input. The sessions were held in a meeting room at the orthopaedic centre and each lasted 2 h. All sessions were audio recorded, with the consent of all participants. At the start of the first session, written informed consent was obtained from all the participants.

##### 2.3.2.1. Session 1: Structure and analysis

The first session started with a round of introductions of the participants, an explanation of the aim of the focus-groups sessions and a summary of results from the previously held interviews (van der Horst et al., 2019). Participants were given the opportunity to react and if needed add new information to this summary. Subsequently, participants were asked for their needs and wishes for an intervention, by writing their ideas on a post-it and sticking it on one of four white posters: pre-operative period, hospital stay, post-operative period and general input for content of the intervention. The session was concluded with a plenary conversation, discussing the generated input and asking for any additions.

After the session, the audio-recording was transcribed and coded using thematic analysis by two authors (AH and SK), to create a first, online version of the intervention for the second focus-group session.

##### 2.3.2.2. Session 2: Structure and analysis

During the second focus-group session there was a summary of the input from the previous session and an opportunity to add extra information. Some small additions were mentioned (e.g., not only a video of the operating theatre, but also of the nursing ward), which were noted and processed.

In line with the input from the interviews, the first focus-group session and previous research on chronic pain (e.g., Trompetter et al., 2014; Veehof et al., 2016) participants were presented with a set of proposed modules for the intervention: information on the spinal conditions and surgery; preparation before surgery and practical tips; how does pain work; pain medication; physical guidelines; recovery and complications; experiences of previous patients; contact details of the hospital; positive psychology exercises; mindfulness exercises; reflection exercises on value-based activities (ACT). For an overview of these modules and more information on their content, see Supplementary Table C1 in Appendix C.

Participants were asked for their opinion on the content of the modules, the timing and time span, and whether modules could be merged or deleted.

**Figure 2:**
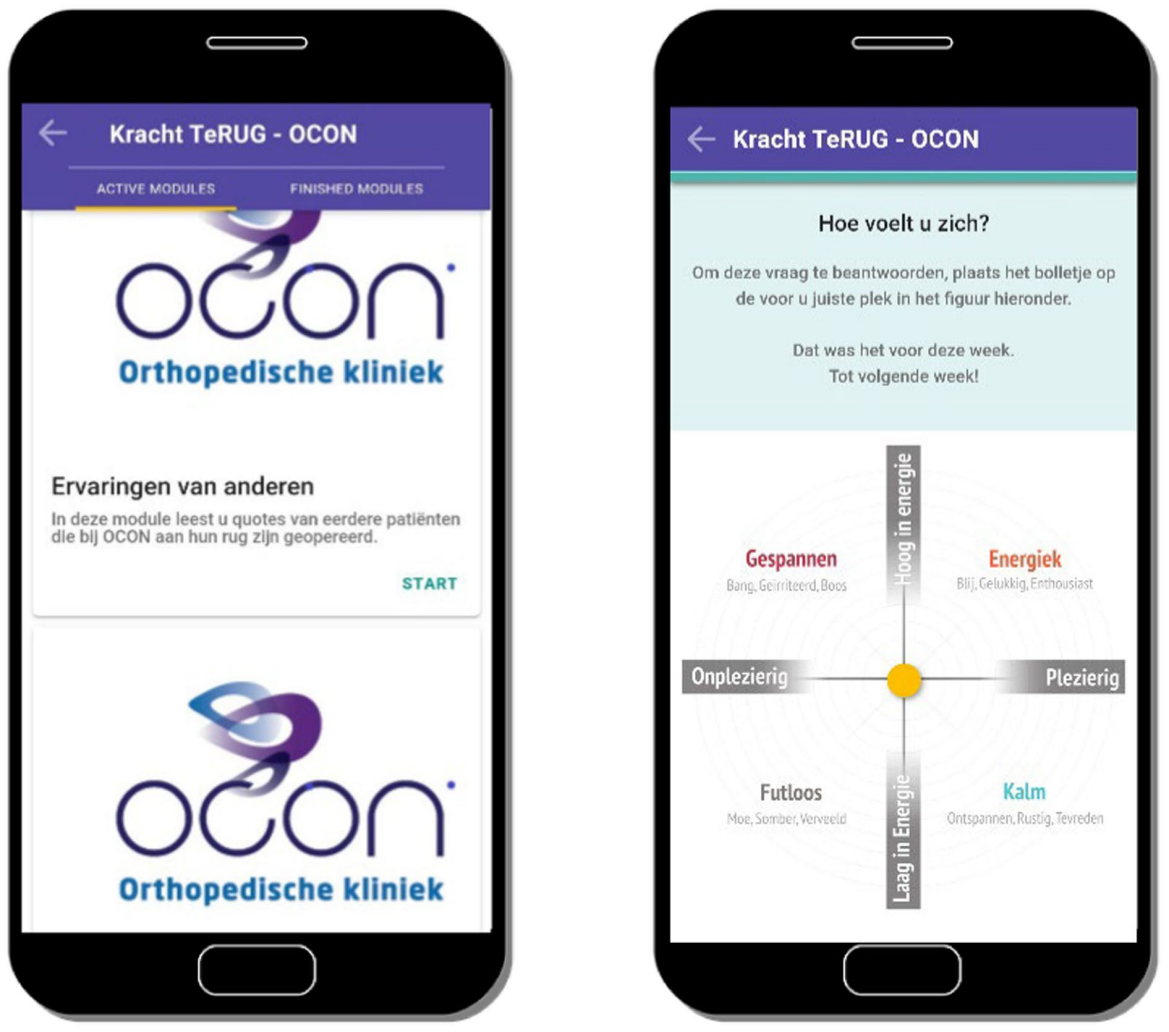
Screenshots of the first version of the intervention Strength Back showing the start screen of the module on experiences of previous patients (left) and showing a question about the current emotional state of the participant (right).

Next, a first online version of the intervention was shown on a screen. This online version was created by using The Incredible Intervention Machine (TIIM). TIIM is an application for IOS and Android, created by the BMS lab from the University of Twente. This software enables researchers to collect participant data and to present them with stimuli or measurement items (e.g., interventions or questionnaires), through smartphone use *via* the TIIM app. TIIM offers both a frontend (what the user sees) in the form of the TIIM app and a backend (what the researcher sees, including a preview of the participant view). The backend, specifically the preview, was used to show the participants the first, online version of the intervention (see Figure 2), during the second focus-group session. They were then encouraged to respond to this.

Subsequently, participants were divided in pairs. Per pair, participants were given a stack of cards with one module shown on each card and several blank cards. Participants were then given three assignments: firstly, to put these cards in an order that matched their preference, stating which module should be displayed when (e.g., before, during or after hospitalisation). Secondly, participants were encouraged to state if they wanted modules to be merged or deleted, and thirdly, if they had any ideas for additional modules (i.e., on the empty cards). Two pairs consisted of a patient and a nurse and due to lack of extra patients, the advanced nurse practitioner and the orthopaedic surgeon both arranged the cards individually. Participants were accompanied by a research-assistant who audio-recorded the discussion and made notes. The session was concluded with a plenary conversation, discussing the generated input from the individual pairs and asking for any additions.

After the session pictures were taken of the way module-cards had been arranged, including any newly added modules. These pictures were summarised and used to create a new intervention prototype for the third session in TIIM. In addition, the audio recording of the session was transcribed verbatim and analysed thematically by one author (AH).

##### 2.3.2.3. Session 3: Structure and analysis

The third focus-group session started with a summary of the second session, to check whether the input had been analysed in line with the view of the previously present participants. New participants were encouraged to react to the summary and to add information, whenever they felt this was needed.

Then, participants were divided in pairs and accompanied by a research-assistant who audio-recorded the discussion and made notes. The patient and professional were given a mobile phone or tablet with the TIIM app on it. In the TIIM app, the intervention ‘Strength Back’ was shown. For screenshots of this version, see Appendix E. The pair was asked to click through the intervention, read the modules and complete some exercises. The research-assistant was given a script with an overview of all modules and which questions to ask the patient and professional while they were looking through the intervention (e.g., what do you think of this module? Is the information clear? Do you like the lay-out?). The following modules were included in the intervention shown to the pairs: introduction; information on spinal conditions and surgery; preparation at home and practical tips; how does pain work; pain medication; physical guidelines; experiences of previous patients; value-based exercises, mindfulness exercises; recovery and complications and contact with the hospital.

The session was concluded with a plenary conversation, discussing the generated input from the individual pairs and asking for any additions. After this third session, participants received a box of chocolates to thank them for their time and effort.

After the session the notes of the research assistants who accompanied the participants during their testing of the intervention, were collected and organised. These notes were summarised. In addition, the audio recording of the session was transcribed verbatim and analysed thematically by one author (AH). The output from the notes and the audio recording was combined to create a new prototype of the intervention in TIIM. The content of this intervention, “Strength Back,” is described in the final paragraph of the results section and was tested in a feasibility study, the results of which will be discussed in a separate article.

### 2.4. Reflexivity

The interviews and focus group sessions were held and led by one author (AH). This author is a female psychologist, trained in interview techniques and in performing qualitative research. The author was a PhD Candidate at the time of the research. The current study was part of a larger PhD research project and this was stated in the information letter that all participants received before the start of the study. The author was familiar with the orthopaedic centre and the professionals that worked there. This also meant the author was familiar with the topics related to the process of spinal surgery and the challenges patients faced in the perioperative phase. The patients that were included in the focus group sessions were new patients, who did not participate in the previous interviews. Therefore, the author had no previous acquaintance with any of the patients that were included in this study. All data analyses were discussed with another author (SK) who is a female associate professor, specialized in eMental Health and engagement with eHealth.

### 2.5. Ethics

This study was approved by the Ethical Committee of the University of Twente (no. 190068). This study was carried out in accordance with the Code of Ethics of the World Medical Association (Declaration of Helsinki). Informed consent of participants was collected in writing and according to good clinical research practice guidelines. Data was pseudonymised as soon as possible and stored in the Cloud Drive of Saxion University of Applied Sciences to protect data files against loss, damage, unauthorised access and digital aging.

## 3. Results

### 3.1. Interviews

#### 3.1.1. Participants

Participating patients (*N* = 12) were equally divided in gender and ranged in age between 45 and 85. All participants had undergone surgery in the previous 3 to 9 months. Educational level ranged from low (*n* = 3) to middle (*n* = 4) and high (*n* = 5). Half of the group were retired, one participant received a disability pension, one worked less than 20 h per week and 4 participants worked for more than 20 h per week. Detailed characteristics of the patients can be found in Supplementary Table A1 in Appendix A and those of the professionals can be found in Supplementary Table A2 in Appendix A.

The group of participating professionals (*N* = 9) included orthopaedic surgeons (*n* = 2, both male), a physical therapist (*n* = 1, male), an advanced nurse practitioner (*n* = 1, female) and nurses (*n* = 5, all female). Years of experience ranged from 5 to 20 years and the majority of the professionals worked more than 20 h per week.

#### 3.1.2. Interviews with patients

After thematic analysis of the patient interviews, the following main themes were identified: content; requirements; timing (before and after surery) and time span of the intervention; use and perceived added value; mental exercises; support or guidance; proposed procedure. See Table 1 for all themes and illustrative quotes of patients, subthemes are described in italics below.

**Table 1:** Thematic analysis – patient interviews – themes and illustrative quotes.

Theme	Subthemes	Illustrative quotes
Content	Advice and practical tips	PT4: *“Practical tips. Such as a “helping hand” < aid > to pick something up from the ground. Things like that.”* – subtheme advice and practical tips
	Physical therapy exercises	PT3: *“Physical therapy exercises, perhaps even with little videos, if that is possible. That would be very valuable.”* – subtheme physical therapy exercises
	Procedure and medical information	PT6: *“Before surgery, I want to know what to expect. I have looked up a lot of information online. It is nice to be able to read and re-read it in an app.”* – subtheme procedure and medical information
	Frequently asked questions	
	Forum	PT2: *“I have read experiences of other people, online. But that’s the problem, only people with negative experiences go online to post their story. You do not find the success stories online.”* – subtheme forum
	Experience stories	PT2: *“If there would have been a story from a previous patient, stating that he or she did not feel any progress the first month either… that would have helped me. That would have comforted me.”* – subtheme experience stories
Requirements	Clear language	
	Easy to use technology	PT2: *“Easy, clear and short. It should be accessible, also for people that are not highly educated, it should be easy to use. Yes, it should be for everyone.”*- subtheme easy to use technology
	Reminders and notifications	
Timing and duration		PT8: *“When it is your first spinal surgery, I think it is good to be able to use the app a few weeks before surgery. It might empower or calm people.”*
Use and perceived added value		PT10: “*You see all kinds of obstacles in your way. That is why I think an app is very good, when you feel insecure, that you can look things up. I do not know exactly what the app would look like, but I would have been happy with it, I think.”*
Mental exercises	Mindfulness exercises	PT6: “*To…yes…just function normally, that I could cook again and do my household chores… do things myself. So yes, I do think that is important, how do you cope with that, and what are your goals after surgery?”* – subtheme value-based exercises
	Value-based exercises	
Support or guidance		PT2: “*I want to get better, I do not need someone to cheer me on, it is my responsibility. I want a programme that enables me to let my body recover. I need to feel it for myself, I need to see it, that is why I would need an app, to do it for myself and by myself.”*
		PT7: *“When the app is clear, why would I need any guidance? Then the app IS the guidance, right?”*
Proposed procedure		PT9: *“Sounds good. That there is sufficient contact. It does meet a need. During recovery, you sometimes feel that you need to solve everything yourself.”*

The suggestions for intervention *content* included the subthemes: advice and practical tips; physical therapy exercises, procedure and medical information; frequently asked questions; forum; experience stories. The subtheme *advice* mainly focused on rest, listening to your body and following hospital, physical guidelines. Although some patients stated they had received enough information from the doctor and in the paper brochure, the majority of patients stated they would have liked to be able to read and re-read *procedural and medical information* in a digital intervention. When the researcher suggested to include some sort of contact or experiences from previous patients in a digital intervention, all patients were very clear that a *forum* should not be included. After suggesting a forum, the suggestion for *experience stories*, collected by the researcher from real, previous patients from the same clinic was mentioned. This got a positive response from half of the participants.

When asked what *requirements* a digital intervention should meet, patients mentioned *clear language*, *easy to use technology* and *reminders and notifications.*

Concerning *timing and duration*, patients agreed that the intervention use should start before surgery, with a focus on providing information, and continue after surgery, to serve as a guide during recovery. The start should be 3–4 weeks before surgery, once the surgery date is known. The intervention should continue 2–3 months after surgery, to make sure there is still some sort of support when picking up daily routines and starting the physical therapy rehabilitation process.

The majority of the patients (*n* = 10) would have *used* the intervention, if it had been available during their own recovery. These patients would have welcomed the intervention as an *added value* to the care they received, stating that it would have been nice to have information at hand, at any time and to feel support and reassurance during recovery.

Most patients (*n* = 9) were open to the idea of pain-related *mental exercises* in the intervention, but the general first association with “exercises” was physical exercises. Some patients specifically wanted to do physical exercises after surgery, whilst other patients, whose pain was gone because of the surgery, did not feel the need for mental exercises. The patients wanting mental exercises to be included in the intervention, felt it would have been helpful, especially at moments when they felt “*overwhelmed*” (PT10) or to take time “*to look at what you feel and how it feels*” (PT2). Exercises where patients would be asked to focus on the pain, e.g., *mindfulness exercises* as a body scan, produced mixed feelings among some patients. Participants did not want to become too negative, focusing on pain, and preferred distraction, whilst others felt facing the pain might enable them to cope with it in a more effective way. Some participants were hesitant about *value-based exercises*, not seeing the need for them, when undergoing something physical as a spinal surgery, where others felt it was important (e.g., see quote PT6 in Table 2).

**Table 2:** Content of the intervention Strength Back.

Module title	Module content	Source of the content
Introduction	Information about the function and content of the intervention	Created by researcher
Information condition	Information about stenos or spondylolisthesis	Paper hospital brochure
Information surgery	Information about surgical procedure	Paper hospital brochure
Physical guidelines	Physical guidelines per week on what to do and not to do after surgery	Paper hospital brochure
Contact the hospital	List of complications when to contact the hospital	Paper hospital brochure
	Telephone number and email address of the hospital	
Videos	Video of virtual tour through the nursing ward	Input during focus-groups, provided by the hospital (orthopaedic surgeon)
	Video of virtual tour through the operating theatre	
Pain medication	Information about types of pain medication, their function, side effects and tapering of medication	Input during focus-groups, provided by the hospital (anaesthesiologist)
Mindfulness exercises	Audio mindfulness exercise – mindful breathing	Based on ACT, created by researcher
	Audio mindfulness exercise – body scan	
How does pain work?	Pain education module	Based on ACT, created by researcher
Physical therapy	Information about physical therapy before and after surgery	Input during interviews and focus-groups, provided by hospital (physical therapist)
	Physical criteria for discharge from hospital	
Practical tips	Tips from previous spinal surgery patients	Input during interviews and focus-groups, created by researcher
Quotes from others	Quotes from previous spinal surgery patients	Input during interviews and focus-groups, created by researcher
Recovery	Text and quotes on the ups and downs during recovery	Based on input from interviews, focus-groups and ACT, created by researcher

All patients pointed out they would not need any *guidance* while doing the exercises and using the intervention, other than technological *support* for the intervention or contact details of the hospital should they have any medical questions or concerns.

During the interview, participants were asked for their response to *a proposed procedure* in which participants would start working with the digital intervention independently before surgery. During the hospital stay a nurse would be available for any help needed and, during the entire use of the intervention, there would be frequent email contact between the participant and a nurse. All patients reacted positively to the proposed design, especially the frequent contact with a nurse.

#### 3.1.3. Interviews with professionals

The following themes were identified in the professional interviews: content; requirements (for professionals and for patients); timing and duration of intervention use; feedback on proposed procedure, supporting patients; impact on own profession.

Concerning *content*, the professionals agreed with the patients on including procedural information, physical guidelines and experience stories. Nonetheless, the professionals also had their own, unique input, different from the patients. The topics *expectation management*; *pain, pain coping and pain medication*; a wish to *screen patients* for any psychological risk factors and a clear *focus on visual content* instead of too much text, were new input and emphasised by several professionals, including both doctors and nurses.

Where patients clearly asked for as much *physical therapy* information and exercises as possible, the physical therapist that was interviewed was not in favour of providing exercises either before or after surgery in the intervention. There would be little use of doing exercises before surgery, as previous physical therapy had not had the desired effect, otherwise patients would not be having surgery. And after surgery, both during admission as in the first few weeks at home, rest and “normal” movement was important to heal. When exercises were possible (light exercises at first and later during rehabilitation), they should be tailor made, accommodated to suit the individual patient by a physical therapist in person, and not provided in general wording in a generic intervention.

*Additional ideas* from the professionals included videos of the operating theatre and nursing ward, providing links to good quality, informative websites and a list of types of professionals you could meet during hospitalisation, possibly accompanied by interviews with them.

The professionals made a distinction between *requirements* for patients and requirements for professionals. *For patients* the intervention should be easy to use, user-friendly, there should be a contact person for help and the language used should be clear and easy to understand for everyone. The intervention should be visually attractive and should also contain as much visual information as possible. It should be accessible on not only a computer, but also, or mainly, on a smartphone or tablet. For the participating professionals clear communication was important and most of their requirements related to implementation. All disciplines should be notified that the intervention has been introduced, the content should be well-known to all, benefits of using the intervention should be provided, there should be enough time for preparation and for guiding intervention use. The intervention should be compact, fit in the current care process and contain information that is congruent to current hospital protocols and guidelines. The intervention should be self-explanatory and patients should be informed and able to use the intervention before admission to the hospital on their own device.

Concerning *timing*, all professionals agreed with patients that the intervention should be available a month before and longer after surgery. All professionals linked the *duration* of the intervention to the length of the recovery process and several existing, contact moments with the hospital, resulting in a postoperative use of the intervention of 6 weeks to 2 or 3 months, depending on the type of surgery.

The opinions of the professionals were divided as to who should *support the patients* during their use of the intervention: nurses or the physical therapist together with a nurse practitioner. The majority of the nurses were open to the idea of supporting patients during their use of an intervention, either on the ward or remotely when the patient is at home.

An intervention could *impact their profession*, by enhancing the daily work of a professional by enabling them to refer patients to written and visual information in the intervention, resulting in well-prepared patients with more peace of mind and fewer questions, with better time investment for professionals. Also, an intervention is a source of uniform information, for both patients and new professionals.

Similar to patients, the professionals were asked for their response to *a proposed procedure*. Professionals were open to the idea of this proposition, but also noticed that the nurse might not be the best professional to give guidance during the entire intervention use, as professional input from a physical therapist might also be required. In turn several nurses pointed out they liked the idea of keeping in contact with patients and that email was a time-efficient way of doing this.

### 3.2. Focus-groups

#### 3.2.1. Participants

During the three focus-groups sessions, several patients and professionals contributed to the design process, by attending one or more sessions. An overview of all participants is shown in Appendix B.

In sum, of the patients (*N* = 7) 5 were female and 2 were male, ranging in age between 35 and 85. One patient visited all three sessions, while the others either visited one (*n* = 4) or two sessions (*n* = 2).

The group of professionals (*N* = 8) included an orthopaedic surgeon (male), a physical therapist (male), an advanced nurse practitioner (female), a research coordinator (female), a team leader nursing staff (female) and three nurses (all female). Professionals ranged in age between 35 and 55. The nurse practitioner and two nurses were present at all three sessions, the orthopaedic surgeon and research coordinator at two sessions. The specialised physical therapist and the team leader of the nursing staff were both present at one session.

#### 3.2.2. Focus-groups – Session 1

The summary of the input from the previous interviews with patients and professionals evoked an emotional response in several patients at the focus-group session. They stated this was a correct and clear representation of their own experience and that they had been unaware that others had felt the same way. No further additions or suggestions were made by the participants to add to this summary.

In all four categories (general, pre-operative, hospitalisation and post-operative) suggestions were collected on the *content* of the intervention (e.g., procedural information) and on *advice* participants had for future patients (e.g., follow guidelines, listen to your body). Also, the *aim* of the intervention was discussed: providing information, for realistic expectation management and providing support during recovery at home. In Supplementary Table D1 (see Appendix D) a summary is given of the post-it notes that were collected during the session.

#### 3.2.3. Focus-groups – Session 2

Based on the first focus-group, several module themes were developed: information on the spinal conditions and surgery; preparation before surgery and practical tips; how does pain work; pain medication; physical guidelines; recovery and complications; experiences of previous patients; hospital contact details; positive psychology exercises; mindfulness exercises; reflection exercises on value-based activities (ACT). These modules were presented to patients and professionals, during the second focus-group session.

During the discussion of these proposed modules, patients were at first hesitant about the module “experiences of previous patients.” However, while discussing the possible content of this module they became enthusiastic and felt it could have added value. The suggested psychological exercises were seen as something positive, to help patients through the ups and downs of the recovery period and the mindfulness exercises to help patients relax in moments of pain, both before and after surgery. One of the professionals stated that the focus on the physical as well as the mental aspect of recovery, was an eye-opener for him.

When the prototype was shown, the name of the intervention was discussed and the option of adding a chart to the intervention, in which people could track their activity level and recovery, including a baseline before surgery. Other topics were also discussed, such as when to contact the hospital or a GP, what the content of the module pain education should be (what medication do you receive and when should you use which one?). Advice on iron, protein and fibre-rich food during recovery, mentioned during session 1, was now regarded as not essential.

During the discussion in pairs, participants were asked to state the order in which they wanted to receive the modules and state whether modules could be removed, added or merged. Except for one pair wanting to remove two modules and combine two other modules, all other pairs wanted to maintain all modules as suggested. Concerning the timing of the modules, all participants indicated that the same modules should start before surgery (i.e., mainly the information modules). However, participants were not unanimous about the timing of the more psychological, supporting modules with either the mental exercises or experiences from previous patients. Some participant pairs stated this type of module should start before surgery, when patients are preparing themselves for surgery, while other pairs stated this should be presented at the time they felt this information was most needed: during the fluctuating process of recovery, thus after surgery.

#### 3.2.4. Focus-groups – Session 3

During the summary of the previous session, no additional information was given by participants. The discussion of the different modules in pairs yielded various textual suggestions (e.g., clarifying certain concepts; shortening or expanding certain sections). All modules were regarded as relevant.

In addition, asking professionals about their needs concerning the implementation of the intervention in their daily working routine, yielded the same information as in previous sessions: professionals needed a clear presentation of the intervention before implementation and the intervention should contain unambiguous information, clear and known to all professionals.

#### 3.2.5. Observation during the sessions

During the sessions, there was considerable interaction between patients and professionals, exchanging information and experiences. Several professionals made notes of what patients mentioned, to put into practice the next day, prior to the development of the intervention.

### 3.3. The intervention “Strength Back”

Based on the interviews and focus-group sessions, the modules displayed in Table 2 were integrated into the final prototype of the intervention “Strength Back.” Certain modules (i.e., *information on spinal condition; information surgery; physical guidelines; contacting the hospital*) were based on information previously provided by the hospital in the form of a paper brochure. Other modules (i.e., *how does pain work; mindfulness exercises*) were based on ACT and created by the research team.

In addition to the modules shown in Table 2, which are available to participants at all times during their recovery process, weekly modules are also provided. As the recovery duration differs, a decompression version of the intervention contains 6 weekly, post-operative modules, while a spinal fusion version contains 12 weekly, post-operative modules. Both versions start 4 weeks before surgery, with three weekly, pre-operative modules. The weekly module contains information that is relevant at that time (e.g., physical guidelines for that week or quotes from previous patients about that period) and a mindfulness exercise and/or a psychological exercise. The PP and ACT exercises included in the weekly modules are shown in Table 3.

**Table 3:** Overview of positive psychology and ACT exercises in the intervention Strength Back.

Name of exercise	Content of exercise	Timing of exercise
Mindfulness exercise*	Mindful breathing and body scan	PRE1** + POST2/POST9***
Wish question	What if you could make a wish for your health? What would this change? And how can you already make a step in that direction today?	PRE1 + POST2/POST3
What makes the surgery worthwhile?	What are value-based activities to do (again, after surgery) or keep doing?	PRE2 + PRE3
A letter to yourself	Write a letter to encourage yourself in hard times, e.g., when recovery is tough	PRE2 + POST2/POST8
Positive statements	Formulate statements to encourage yourself during a hard time, e.g., when recovery is tough	PRE2 + POST4/POST5
Valuable image	Which picture (on your mobile phone) shows what you find important and valuable, how can you use this during recovery (e.g., as a goal)?	POST3/POST4 + POST10
Three positive things	Which three things made you grateful today and what was your own part in this?	POST5/POST7
Conscious enjoyment	Which activities have you undertaken today that often go unnoticed, but which, today, you have consciously engaged in and enjoyed? E.g., cooking, taking a shower or walking?	POST5/POST6 + POST11

*Mindfulness exercises are also constantly available in a separate module; **PRE, weekly module before surgery; POST, weekly module after surgery; ***Timing for decompression/spinal fusion version.

## 4. Discussion

This study describes the developmental process of “Strength Back,” a digital health intervention for spinal surgery patients. In this study interviews and focus-groups were conducted to determine important needs and relevant contextual factors. The results were used to develop an app with psycho-educational, ACT-based and positive psychology interventions. Design of the digital intervention was also discussed with patients. Based on the interviews and focus-groups the following components of the app were developed: information about spinal conditions, surgery, physical guidelines and physical therapy; patient stories; a pain information module; psychological exercises. We will discuss these modules in the context of the core aim of the intervention, namely to increase psychological flexibility.

*Information on spinal condition, surgery* and *physical guidelines* was deemed essential by both patients and professionals and was therefore included in the intervention. These information modules served as valuable, practical information, aiming at realistic expectation management as unrealistic or unfulfilled expectations about surgery and preoperative stress may lead to experiencing higher levels of postoperative pain (Iversen et al., 1998; Munafo and Stevenson, 2003; Arpino et al., 2004; Granot and Ferber, 2005; Morone et al., 2010; Mancuso et al., 2016, 2018).

Patients asked for as much *physical therapy* information and as many exercises as possible. However, the physical therapist stated it was not desirable to provide generic exercises. He was in favour of tailor-made exercises, provided by a physical therapist in person, instead of through the online intervention. In line with this, the Fear Avoidance Model (Vlaeyen and Linton, 2012) and the Avoidance-Endurance Module (Hasenbring and Verbunt, 2010) underline the importance of a personalised approach. A physical therapist might recognise avoidance or endurance tendencies and can act accordingly to ensure the patient retains a good rest-activity balance. Psychological flexibility is important in this context, as it enables a patient to pursue value-based activities instead of fighting, avoiding or controlling the pain (Vowles et al., 2014). This focus on value-based activities and psychological flexibility can be used in personal physical therapy sessions. Therefore, it was decided not to include physical therapy exercises in the intervention. Instead, information on physical therapists in the region of the hospital was provided in the intervention.

The module on *quotes from previous patients,* contained experience stories that patients regarded as added value to the intervention. This was provided in the form of brief quotes, categorised per period (pre- or post-operative), substituting the initial idea of having a forum. Similar to the information modules discussed earlier, these experience stories from previous patients were included in the intervention with the aim of forming realistic expectations and motivating users to engage with the app.

Textual information as well as an animation was created for the module on *how pain works*. This is in line with Meppelink et al. (2015). They found that spoken animation is the best way to communicate complex health information to people with low health literacy and does not negatively influence highly literate audiences. It even bridges the information processing gap between audiences with low and high health literacy. The recall differences between the two groups are eliminated, ensuring optimal information uptake by all participants (Meppelink et al., 2015). The animation consisted of drawings and a narrator explaining the function of pain, influencing factors of pain and how to deal with pain after surgery. By providing information on how pain works, including the use of metaphors as is common in ACT, acceptance of pain is targeted (Vowles et al., 2014). Through acceptance and committed action in line with personal values, psychological flexibility can be targeted (Hayes et al., 2012).

The module on *mindfulness exercises* contains two mindfulness exercises, i.e., a body scan and a mindful breathing exercise. Being in the moment is a key element of ACT and an important component of psychological flexibility (Hayes et al., 2012). Therefore, these exercises contribute to the main outcome of the intervention.

The psychological exercises in the weekly modules focused on several elements of ACT. In part, they were based on the online intervention “Living with pain” (Trompetter et al., 2014) which focuses on patients with chronic pain. The exercises supported participants in clarifying their personal values and forming realistic goals (e.g., value-based activities) to commit to committed action during recovery. PPIs may support patients to notice and savour positive experiences (Bolier et al., 2013). This may strengthen their commitment to value-based action. Also, PPIs promote the experience of positive emotions. Positive emotions stimulate people to think in an open, tolerant and constructive way [Broaden and built theory; Fredrickson (2001)]. According to this theory, they provide an opportunity for growth, in an outward looking, flexible and creative manner (*broadening*). This encourages resource *building*, such as forming a social network, which creates an upward spiral for mental well-being and flourishing. Thus, positive emotions build resources that can be drawn on later to improve the odds of successful coping with negative emotions, including pain, leading to resilience and well-being.

The inclusion of PPIs showed the importance of combining bottom-up and top-down strategies. At first the participating patients did not see the value of PPIs. However, there is growing evidence that PPIs promote recovery and adaptation (e.g., Bohlmeijer and Westerhof, 2021). In this study, when experiencing the PPIs, the patients became more enthusiastic about their potential benefits. The professionals in this study, normally mainly focused on the physical well-being of their patients, were open to the idea from the start, stating that they saw the potential and indicated it was an eye-opener to them to focus on positive value-based goals during recovery. This suggests that the combination of stakeholder involvement and scientifically proven input can provide valuable input for a design process, by interacting and reinforcing one another.

Interestingly, both patients and professionals in the interviews as well as in the focus-group sessions indicated they wanted the intervention to start before surgery and last for several weeks or even months after surgery. This is in line with findings in current research expressing the wish of orthopaedic patients, being granted a sense of control and responsibility over their recovery by initiating and using interventions preoperatively (Robinson et al., 2021). Similarly, Scott et al. (2017) found that preoperative introduction of an intervention was superior and led to higher app use than an intervention with merely a postoperative timing. In addition, Robinson et al. uses the term surgical teachable moments to emphasise the potential of captivating the preoperative patient mind-set in encouraging perioperative behaviour change and optimising postoperative outcomes (Robinson et al., 2020). The intervention Strength Back enables patients to prepare for surgery, as well as develop long-term skills to not only cope with the fluctuating recovery process and overcome challenges in recovery, but also to thrive and increase their mental well-being in a sustainable way. This is in line with literature on PP and ACT (Sturgeon and Zautra, 2010) and goes beyond currently available psychological interventions for surgery patients as they mainly focus on the period before surgery (Powell et al., 2016).

The idea of providing procedural information in combination with psychological exercises to develop long-term skills, could also be beneficial for other target populations. Our results showed that patients are intrinsically motivated to read information about their condition and the upcoming procedure. This can be used to trigger them and to provide them with additional information, on mental well-being and sustainable resilience or psychological flexibility to cope with physical complaints, pain and other hardships in their life. Pain education and information on medication might help several other surgical patient groups and the ACT and PP exercises could benefit other target populations who experience stressors or major life events.

Our intervention focusses on long-term skills. We encourage future researchers to focus on more than just surgery preparation or short-term recovery, as surgery is just one event in a patients’ lifetime filled with health-related experiences. Also, the patients in our study were positive about this long-term focus.

Our research shows the importance of combining top-down, scientifically proven input and bottom-up, stakeholder input in participatory design. We encourage future researchers to follow this example and report on the strengths and limitations following this approach.

### 4.1. Strengths of this research

Following the CeHRes Roadmap (van Gemert-Pijnen et al., 2011) was a strength of this research, as it emphasises stakeholder involvement from the start. This was done throughout the design process, with different types of data collection (i.e., interviews as well as focus-groups), combined with an iterative process of continuous formative evaluation in the form of checking our findings with these stakeholders, establishing a true co-creation process. High adherence to the digital intervention is expected due to the involvement of future users in the development of the intervention right from the start. In addition, using this roadmap, as well as describing our method in detail, provides future researchers who aim to develop an intervention with valuable information.

In this study the intervention Strength Back was developed using an existing application, namely TIIM. This provided the research team with a cost-effective, previously tested, functional application and it enabled the participants to focus on the content rather than on the lay-out of the intervention.

Patients as well as professionals, showed considerable enthusiasm and actively participated during the entire developmental process, suggesting they felt engaged in the development process of the intervention and its future purpose. This active participation in the development process shows promise for the implementation and adoption of the intervention in the existing care process by these same professionals.

Even though there are several psychological interventions preparing patients for surgery (Powell et al., 2016), our intervention is unique in its long-term focus after surgery and in combining patient education on medication and pain with elements of positive psychology and ACT. This might allow patients to develop long-term skills for sustainable resilience in the face of (chronic) pain or other hardships in their life.

### 4.2. Limitations of this research

The aim of an equal distribution of patients and professionals in the focus-groups was not achieved. Due to the over-representation of professionals in focus group session 2, there might have been a dominance effect, a halo effect or a social desirability bias. Nonetheless, the patients voiced their needs and preferences clearly and did not seem to be influenced by the presence of the professionals during these sessions. The patients were no longer in care with the healthcare professionals, therefore the effect of dependency was minimised. In addition, patients occasionally voiced different opinions from the healthcare professionals (e.g., about psychological content of the intervention), suggesting they felt safe and free to express their genuine judgement.

Using TIIM could also be a possible limitation of the current research as it has restricted functionalities. This meant that certain stakeholder wishes could not be granted or guaranteed.

Using participatory design meant that data collected during the design process was analysed in a short time-span, ensuring the use of this stakeholder input in subsequent focus-group sessions. This led to a constant balance shift between in-depth analysis and the ongoing design process. The overlapping processes of data collection, data analysis and evaluation with stakeholders, ensured that the essence of stakeholder input was gathered.

As the current research was conducted in a single orthopaedic centre, future research should focus on whether the intervention is also suitable for other hospitals. As the themes and challenges mentioned by patients are largely universal, it is to be expected that the content of the current intervention is quite generalizable and applicable to other surgical patient groups.

## 5. Conclusion

In summary, the current study demonstrates how combining a theory driven focus (i.e., on ACT and PP) with input from a broad range of stakeholders, gathered in several ways (i.e., through interviews and focus-group sessions), can lead to a unique and innovative intervention. Information and quotes of previous patients were included in the intervention to facilitate realistic expectation management. An animation on the working mechanisms of pain was included to convey complex health information and target acceptance of pain. Mindfulness exercises in the intervention focus on being in the moment as this is a key component of ACT and psychological flexibility. The intervention covers both the preoperative as well as the postoperative period and aims to develop long-term skills for sustainable resilience. The intervention developed has the potential to increase psychological flexibility and mental well-being. In turn, this may enhance the recovery process and prevent negative postoperative outcomes such as severe postoperative pain or even chronic pain. The next step is to test the usability and feasibility of the new intervention.

## Data availability statement

The datasets presented in this article are not readily available because the dataset contains personal quotes of patients and health-care professionals. Requests to access the datasets should be directed to AH, a.vanderhorst-1@utwente.nl.

## Ethics statement

The studies involving human participants were reviewed and approved by the Ethical Committee of the University of Twente. The patients/participants provided their written informed consent to participate in this study.

## Author contributions

KS, SK, EB, and AH handled conception and design. AH collected the data. AH and SK analysed the data and wrote the draft-version. KS, EB, and SK supervised and critically reviewed the manuscript. All authors gave approval and agreed to be accountable for all aspects of the work.

## Funding

This study was funded through an educational grant from the Dutch Ministry of Education, Culture and Science, provided by Saxion University of Applied Sciences.

## Conflict of interest

The authors declare that the research was conducted in the absence of any commercial or financial relationships that could be construed as a potential conflict of interest.

## Publisher’s note

All claims expressed in this article are solely those of the authors and do not necessarily represent those of their affiliated organizations, or those of the publisher, the editors and the reviewers. Any product that may be evaluated in this article, or claim that may be made by its manufacturer, is not guaranteed or endorsed by the publisher.

## Abbreviations

ACT: Acceptance and Commitment Therapy
PP: Positive Psychology
PPI’s: Positive Psychology Interventions
TIIM: The Incredible Intervention Machine
