# Supplementary material for: Strength Back – A qualitative study on the co-creation of a positive psychology digital health intervention for spinal surgery patients
Source: Front Psychol. 2023 Apr 21;14:1117357. doi: 10.3389/fpsyg.2023.1117357 (PMC10160468; doi:10.3389/fpsyg.2023.1117357)

Supplementary Material

APPENDICES

**Appendix A – Characteristics of patients and professionals participating in the interviews**

*Table A1. Participant characteristics at time of interview – patients (n=12)*

| Patient | Gender | Age range^1^ (years) | Time since surgery | Relationship | Education level^2^ | Occupational status |
| --- | --- | --- | --- | --- | --- | --- |
| P1 | M | 75-80 | < 6 months | Yes | Middle | Retired |
| P2 | M | 60-65 | < 6 months | Yes | High | Retired |
| P3 | M | 60-65 | 6-9 months | Yes | High | Paid work > 20 hrs. a week |
| P4 | F | 80-85 | 6-9 months | No | Low | Retired |
| P5 | F | 65-70 | < 6 months | No | High | Retired |
| P6 | F | 65-70 | 6-9 months | Yes | Middle | Retired |
| P7 | F | 50-55 | < 6 months | Yes | Middle | Paid work < 20 hrs. a week |
| P8 | M | 50-55 | < 6 months | Yes | Low | Disability pension |
| P9 | M | 55-60 | < 6 months | Yes | High | Paid work > 20 hrs. a week |
| P10 | F | 45-50 | < 6 months | Yes | High | Paid work > 20 hrs. a week |
| P11 | M | 55-60 | < 6 months | Yes | Middle | Paid work > 20 hrs. a week |
| P12 | F | 65-70 | < 6 months | No | Low | Retired |

^1^ To ensure anonymity age ranges instead of exact ages are shown. ^2^ Low: primary and lower secondary education; middle: upper secondary education; high: higher vocational training and university

*Table A2. Participant characteristics at time of interview – professionals (n=9)*

| Professional | Profession | Gender | Age range^1^ (years) | Education level^2^ | Years of prof. experience | Amount of hrs work p/wk. |
| --- | --- | --- | --- | --- | --- | --- |
| OS1 | Orthopaedic surgeon | M | 40-45 | High | 9 years | > 20hrs per week |
| OS2 | Orthopaedic surgeon | M | 35-40 | High | 5 years | > 20hrs per week |
| PhTh | Physical Therapist | M | 35-40 | High | 13 years | > 20hrs per week |
| N1 | Nurse | F | 25-30 | Middle | 5 years | 20 hrs per week |
| N2 | Nurse | F | 30-35 | Middle | 10 years | > 20hrs per week |
| N3 | Nurse | F | 40-45 | Middle | 20 years | < 20hrs per week |
| N4 | Nurse | F | 40-45 | Middle | 14 years | > 20hrs per week |
| N5 | Nurse | F | 30-35 | Middle | 5 years | < 20hrs per week |
| ANP | Advanced Nurse Practitioner | F | 25-30 | High | 5 years | > 20hrs per week |

^1^ To ensure anonymity age ranges instead of exact ages are shown. ^2^ Low: primary and lower secondary education; middle: upper secondary education; high: higher vocational training and university

**Appendix B – Participants of focus-group sessions – patients and professionals**

*Table B1. Focus-group participants - patients*

| Participant | Gender | Age range^1^ (years) | Session 1 | Session 2 | Session 3 |
| --- | --- | --- | --- | --- | --- |
| F-PT1 | F | 80-85 | X | X | - |
| F-PT2 | M | 55-60 | X | - | X |
| F-PT3 | F | 65-70 | X | X | X |
| F-PT4 | F | 35-40 | X | - | - |
| F-PT5 | F | 60-65 | X | - | - |
| F-PT6 | F | 60-65 | - | - | X |
| F-PT7 | M | 75-80 | - | - | X |

^1^ To ensure anonymity age ranges instead of exact ages are shown.

*Table B2. Focus-group participants - professionals*

| Professional | Profession | Gender | Age range^1^ (years) | Session 1 | Session 2 | Session 3 |
| --- | --- | --- | --- | --- | --- | --- |
| F-N1 | Nurse | F | 35-40 | - | - | X |
| F-N2 | Nurse | F | 45-50 | X | X | - |
| F-N3 | Nurse | M | 50-55 | X | X | X |
| F-PhTh | Physical Therapist | M | 35-40 | X | - | - |
| F-OS | Orthopaedic surgeon | M | 45-50 | - | X | X |
| F-ANP | Advanced Nurse Practitioner | F | 30-35 | X | X | X |
| F-RC | Research Coordinator | F | 35-40 | - | X | X |
| F-TL | Team leader nursing staff | F | 35-40 | - | X | - |

^1^ To ensure anonymity age ranges instead of exact ages are shown.

**Appendix C – Overview suggested modules and content during focus-group session 2**

*Table C1. Overview suggested modules and content during focus-group session 2*

| Title module | Suggested content |
| --- | --- |
| Information on spinal condition and surgery | - Information on spinal condition - Information on surgical procedure - Information for family - Video operating theatre |
| Preparation for surgery and practical tips | - Preparation at home, information (e.g. ordering walking aids, nutrition suggestions) - Practical tips from previous patients (e.g. prepare meals and groceries before surgery, transport from hospital to home may be painful) |
| How does pain work? | - Explanation on the working mechanisms of pain in the brain and body - Metaphor tug-of-war (focus on fighting with pain costs energy and shifts the focus from value-based activities) |
| Pain medication | - Explanation of the use of pain medication (incl. reminder from the app to take medication) |
| Physical guidelines | - Physical guidelines on what to do and not to do after surgery - Videos and images of physical therapy exercises - Reminder– are you in line with expected physical guidelines/activities at this point of recovery? If not, what can you do? |
| Recovery and complications | - Explanation that recovery goes with ups and downs, coping with relapse, listening to your body, dealing with expectations - Link to value-based exercises, such as “What makes this surgery worth-while” and “Which activities are valuable to you and can you work towards during recovery?” - Reminders from the app: “How is your rest-activity balance?” |
| Experiences of others | - Experiences of previous patients |
| Contact with the orthopaedic centre | - List of possible complications during recovery, when to call the hospital - Contact details (phone and email) of orthopaedic centre |
| Mindfulness exercise | - Body scan - Mindful breathing |
| Exercises / reflections | - What makes this surgery worthwhile? - Recognise your values - Setting goals and determining actions |

**Appendix D – Input generated during focus-group session 1**

*Table D1. Input generated on post-its during focus-group session 1*

| General | Pre-operative period | During hospitalisation | Post-operative period |
| --- | --- | --- | --- |
| *Content:*  Email contact instead of only telephone no.  Different intervention versions per physical condition and age | *Content:*  Information on medication, insurance, mental and physical strain during recovery (expectation management)  Setting a target for your own recovery  Video operating theatre | *Content:*  Criteria what physical activity level you should reach before leaving hospital  Information for family members (e.g. children, what to expect when visiting parent in hospital)  Information on medication, on available care at home (staff and equipment/aids) | *Content:*  Questionnaire to clarify disabilities before surgery, in order to set goals for recovery  Help/information to re-initiate movements that caused pain and fear before surgery  Information on medication  Automated check from the app checking progress and rest/activity balance, automated feedback  Schedule with physical guidelines per day/week  Advice on iron, protein and fibre-rich food during recovery |
| *Advice to new patients:*  Recovery differs per patient, listen to your own body  Dare to ask medical staff questions, take notes during meetings | *Advice to new patients:*  Arrange to have a bed in your living room, pre-cook meals, put daily-use items higher before surgery to avoid bending over, organise other aids (e.g. raised toilet seat) | *Advice to new patients:*  Follow the physical guidelines, listen to your own body  Try not to have (fixed) expectations about recovery | *Advice to new patients:*  Show hospital information to medical staff at home  Arrange comfortable (pain free) transport from hospital to home (not a regular taxi-minibus)  Dare to move, also make movements you couldn’t do before surgery, but not too much at once, follow guidelines |
| *Aim of intervention:*  Support for patients: schedules and information  Source of unambiguous information for all (new) medical staff |  |  | *Aim of intervention:*  Support for transition from (safe) hospital to (insecure) home, both mentally and physically |
| *Practical:* Help patients install the application/intervention | | | |
| *Timing:* Start from screening appointment/when surgery date is known, 2-3 months before surgery  Last until 2-3 months after surgery | | | |

**Appendix E – Screenshots of the intervention Strength Back (“Kracht TeRUG”)**

*Screenshot of the information on the spinal condition, the animation on pain education and the video of the nursing ward*


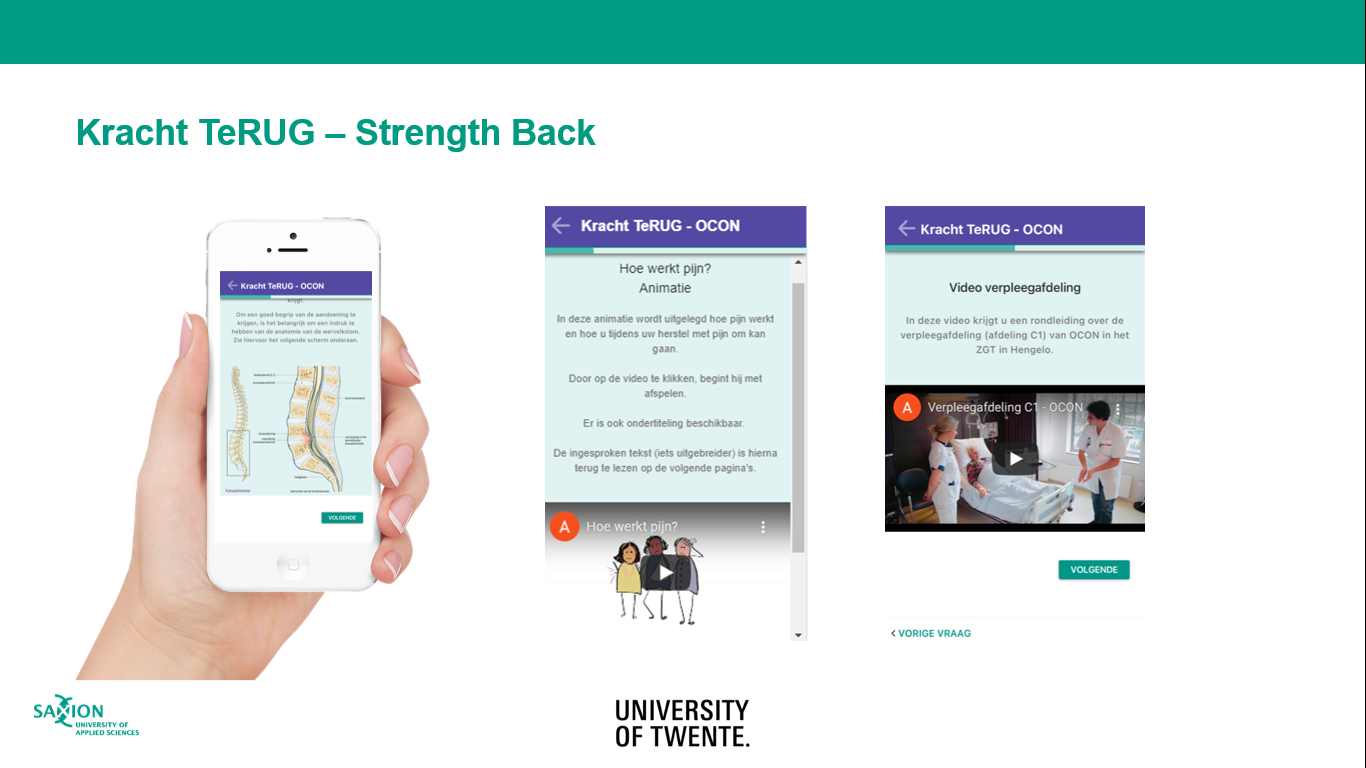


*Screenshot of the ‘wish question’, text about acceptance of pain and “mindful breathing” exercise*


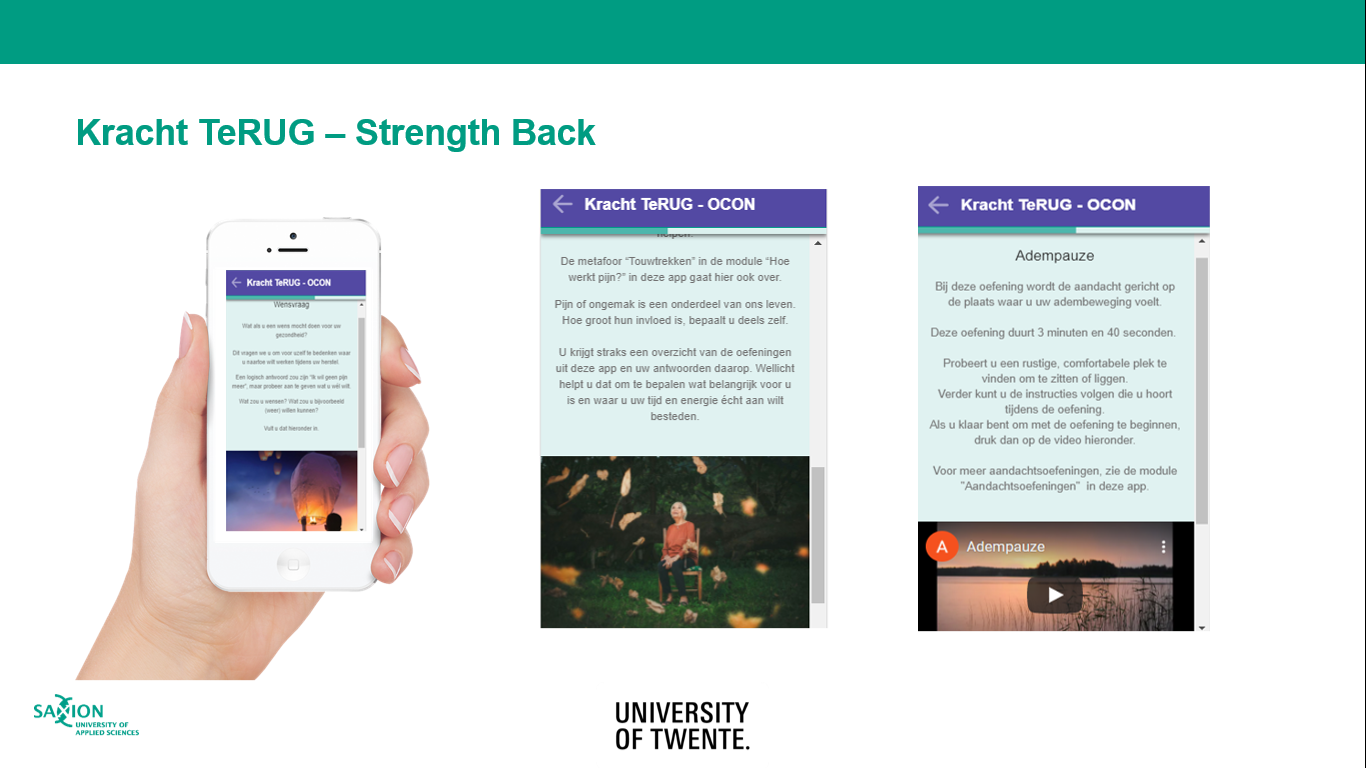

Supplement: Supplementary file 1 [file Data_Sheet_1.docx]
